# Supplementary material for: A Likelihood Approach for Real-Time Calibration of Stochastic Compartmental Epidemic Models
Source: PLoS Comput Biol. 2017 Jan 17;13(1):e1005257. doi: 10.1371/journal.pcbi.1005257 (PMC5240920; doi:10.1371/journal.pcbi.1005257)
Supplement: S1 File — (TAR.GZ) [file pcbi.1005257.s014.tar.gz › HSPH_Online-SI-Revision/Additions/IPoi10/n100-extreme_noisy/n100-extreme_noisy_BENCH2_summary.pdf]

peak -8	3w spec	3w cum	5w spec	5w cum	end	3w spec LOG	3w cum LOG	5w spec LOG	5w cum LOG	end LOG
mean	87.3	89.1	78.8	83.5	37.1	1.5	1.4	1.9	1.7	1.1
median	95.4	96.4	89.5	96.9	26.7	1.3	1.2	1.4	1.4	0.3
quantile .1	59.2	61.2	44.7	53.3	2.5	0.3	0.4	0.2	0.3	0.
quantile .9	99.4	99.2	99.9	99.7	78.2	2.9	2.5	3.7	0.3	2.9
20	20	16	18	10						

peak -4	3w spec	3w cum	5w spec	5w cum	end	3w spec LOG	3w cum LOG	5w spec LOG	5w cum LOG	end LOG
mean	39.6	45.8	25.	32.9	10.8	0.3	0.4	0.2	0.3	0.
median	35.7	42.	20.8	29.2	7.4	0.2	0.3	0.1	0.2	0.
quantile .1	14.4	24.1	12.2	12.	2.	0.1	0.1	0.1	0.1	0.
quantile .9	73.2	73.2	40.9	62.7	23.5	0.8	0.8	0.3	0.1	0.1
28	32	33	27	30						

peak	3w spec	3w cum	5w spec	5w cum	end	3w spec LOG	3w cum LOG	5w spec LOG	5w cum LOG	end LOG
mean	40.	24.1	77.7	29.3	37.8	0.2	0.1	0.2	0.1	0.2
median	33.8	24.1	50.5	28.3	33.3	0.2	0.1	0.2	0.1	0.1
quantile .1	23.3	13.3	35.9	17.8	26.4	0.1	0.1	0.1	0.1	0.1
quantile .9	72.4	36.	159.1	41.	56.2	0.3	0.2	0.4	0.1	0.2
43	49	39	46	43						

peak +4	3w spec	3w cum	5w spec	5w cum	end	3w spec LOG	3w cum LOG	5w spec LOG	5w cum LOG	end LOG
mean	152.8	106.	276.5	118.9	135.1	0.3	0.3	0.4	0.3	0.3
median	77.7	62.2	106.	70.5	85.8	0.3	0.2	0.3	0.2	0.3
quantile .1	38.9	36.1	41.6	37.	38.4	0.2	0.1	0.2	0.1	0.2
quantile .9	318.5	239.	536.3	251.6	266.5	0.5	0.5	0.7	0.1	0.5
27	30	23	29	28						

peak +8	3w spec	3w cum	5w spec	5w cum	end	3w spec LOG	3w cum LOG	5w spec LOG	5w cum LOG	end LOG
mean	402.6	291.4	532.9	315.	333.7	0.4	0.4	0.5	0.4	0.4
median	144.4	109.	156.4	116.	135.7	0.3	0.3	0.4	0.3	0.3
quantile .1	42.2	40.9	44.6	41.1	41.6	0.2	0.2	0.2	0.2	0.2
quantile .9	589.8	490.1	1043.1	522.5	587.7	0.8	0.7	1.	0.2	0.8
22	25	22	24	22	{}	{}	{}	{}	{}	{}
